# Integrated Community Case Management of Childhood Illness: What have We Learned?

**DOI:** 10.4269/ajtmh.94-3intro2

**Published:** 2016-03-02

**Authors:** Bernadette Daelmans, Awa Seck, Humphreys Nsona, Shelby Wilson, Mark Young

**Affiliations:** Department of Maternal, Newborn, Child and Adolescent Health, World Health Organization, Geneva, Switzerland; Health and Nutrition Section, The United Nations Children's Fund, Ouagadougou, Burkina Faso; Integrated Management of Childhood Illness, Ministry of Health, Lilongwe, Malawi; Integrated Delivery, Bill & Melinda Gates Foundation; Child Health Unit, Health Section, The United Nations Children's Fund

The evaluations of integrated community case management (iCCM) of childhood illness in Ethiopia, Malawi, and Burkina Faso published in this issue provide important new information to guide program design and implementation. Recognizing that in most countries with a high burden of child mortality, access to health services is limited for many families and their children, the World Health Organization (WHO) and the United Nations Children's Fund (UNICEF) identified iCCM as an effective evidence-based strategy to increase coverage of lifesaving interventions and reduce preventable child deaths.^1^ Few program evaluations of iCCM at scale exist.^2^ The reports therefore are unique and valuable. However, none of the three reports demonstrated the desired iCCM objectives of increasing care seeking for childhood illness and improved coverage of effective treatment interventions at the population level.

Although the results of these three studies are humbling, they provide a new impetus to analyze prerequisites for successful iCCM implementation at national scale. By early 2015, 47 of 75 countries accounting for the highest burden of maternal and child mortality had adopted a national policy allowing community health workers to treat childhood conditions.^3^ In the transition from the Millennium Development Goals to the Sustainable Development Goals, countries are revisiting their progress and making strategic choices about how to increase access to essential child health services. For countries with a high burden of child mortality, new funding opportunities are available including the Global Fund to Fight AIDS, Tuberculosis and Malaria (GFATM) and the Global Financing Facility. It is therefore a public health imperative to generate and synthetize evidence of best practices for implementation of community health worker (CHW) programs.

As partners who assisted governments in the introduction, scale-up, and periodic review of iCCM implementation in Burkina Faso, Ethiopia, and Malawi, we have summarized in Table 1 key characteristics of policy and implementation in each country. Using a set of well-established benchmarks for iCCM implementation,^4^ we present four key messages to consider for future action.

First, when introducing iCCM, governments should lead and support implementation by adopting a national policy and ensure that iCCM is well integrated and costed within the national health sector plan. Community-based health interventions are not a panacea for a weak health system, nor can CHWs function in isolation from the health system. In Burkina Faso, government concerns about feasibility and sustainability of iCCM scale-up prevented bold decisions on the selection of CHWs. As a result, pilot implementation involved community health volunteers who were often elderly and illiterate and did not receive any predictable remuneration for providing health services. Their motivation remained low, and there was little evidence of their contribution to increased care seeking. In contrast, iCCM was well implemented in the Health Extension Worker Program in Ethiopia and in the 2006–2010 national health sector program under the Essential Health Care package in Malawi. In both countries, iCCM was introduced with clear visibility and commitment at all levels of the health system, and evidence was generated about the potential of CHWs to treat children in the community effectively.

Second, scaling up of iCCM requires investment in capabilities and system supports at all levels of the health system. iCCM training in Ethiopia and in Malawi followed a set of quality criteria, with emphasis on observed clinical practice and follow-up after training. In Burkina Faso, a rapid model of cascade training with limited attention to clinical practice was deployed. WHO recommends that, for iCCM training, quality criteria should include a low trainer-to-participant ratio of one to four, an overall duration of iCCM training of at least 5 days, clinical practice for at least 40% of the training, and a first follow-up visit for trained CHWs within 6 weeks of course completion.^5^–^7^ In Ethiopia and Malawi, independent surveys demonstrated that trained CHWs were able to provide quality of care that was similar to that of health professionals in outpatient facilities, whereas in Burkina Faso, the quality of care provided to sick children by the CHWs was inadequate. In addition to caregiving capabilities, it is essential to strengthen management skills of health facility providers and district and regional managers to ensure that all necessary health systems supports are in place for community case management, including uninterrupted availability of commodities and regular supportive supervision with case observation.

Third, monitoring and evaluation should be an integral part of iCCM scale-up. In Ethiopia and Malawi, maximum benefit was derived from the presence of an independent evaluation team. Quality of care surveys were conducted in both countries at an early stage of program rollout, and these provided valuable information.^8^,^9^ Indicators for assessing implementation strength, as reported by Hazel and others^10^ in their accompanying commentary, were adopted as part of the iCCM monitoring system in all three countries, but concerns remained about the completeness and reliability of the data. An independent assessment of implementation strength was undertaken in Burkina Faso,^11^ Ethiopia,^8^ and Malawi,^12^,^13^ providing a new perspective on effective methodologies for quality improvement. Annual stakeholder meetings convened by the independent evaluation team fostered a culture of peer learning and demonstrated the importance of partnership in reviewing and analyzing data and in mobilizing action.

Our fourth message relates to the importance of community mobilization and demand generation. Insufficient awareness of communities about the availability of iCCM services was an important finding of the household survey and qualitative interviews in Ethiopia.^14^ In Malawi, the first annual review meetings convened by the independent evaluation team highlighted the importance of community engagement in building and managing a village health clinic. The issue of demand also raises the question of who was targeted. In Malawi, the iCCM scale-up strategy focused on hard-to-reach areas (HTRAs) defined according to well-established criteria, although those criteria were applied differently across districts, so a limitation of the evaluation is that the HTRA could not be mapped. However, the Malawi Millennium Development Goals population survey completed in 2014 showed no increase in care-seeking behavior for common childhood illnesses at the population level compared with the survey conducted in 2010, calling into question assumed knowledge of who really are the families that do not seek care.

Much can be learned from the three country articles presented in this issue of the journal. As argued by Hazel and others,^10^ this is the time for strengthening program efforts and facilitating the right investments. Over 20 countries have included iCCM in national plans that have been approved by the GFATM. In these countries, governments and partners must be guided by best practices for iCCM scale-up. Specifically, the findings call for investment in leadership and health workforce capabilities, with a relentless pursuit of good management.

In all the three countries, results of the new studies have been used for policy dialogue and program strengthening. For example, in Burkina Faso, the government has defined a profile for CHWs eligible to provide iCCM with minimum educational standards, and CHWs will be provided with a stipend. The comprehensive iCCM approach, initially implemented in two health districts, is now being scaled up in remote areas in 22 health districts, and training will be based on quality criteria with due attention to clinical practice. In Ethiopia and Malawi, mentorship in health facilities and peer learning are being implemented as part of follow-up. In Malawi, the use of m-health technology has been introduced to ensure uninterrupted provision of commodities and strengthened clinical case management by CHWs.^13^,^15^,^16^

As countries prioritize iCCM, continued investment in coordination, monitoring, evaluation, and implementation research will be of great value. We need to better understand who are the families and children who access health care the least and how to reach them effectively. We also need to better understand how to build the confidence of CHWs and sustain their motivation to deliver quality child health services, as well as how to increase our ability to assess their contribution to the improvement of child health indicators. Finally, we need to explore the benefits of a more comprehensive approach for caring for newborns and children in the community, by focusing not only on illness but also on providing families with support for home care practices from pregnancy through the first 2 years of a child's life.^5^–^7^

The need to foster a culture that generates and uses data for quality improvement seems to be the most important lesson that we have learned. We hope that the results of evaluations of iCCM implementation reported in this issue of the journal will be a catalyst for countries and partners to strengthen community delivery of essential child health interventions and to invest in real-time monitoring and evaluation of their implementation.

## Figures and Tables

**Table 1:** Key characteristics of iCCM in three countries

	Burkina Faso	Ethiopia	Malawi
Political will	No firm commitment, concerns about feasibility and sustainability	Commitment as part of national health sector development plan	Commitment as part of national health sector plan
Policy	Pilot for proof of concept in two districts	Part of national Health Extension Program	Part of Essential Health Care package
CHWs	Volunteers with low literacy, no fixed remuneration	Government cadre, literate, fixed remuneration	Government cadre, literate, fixed remuneration
Geographical scope	Rural communities in two districts: two trained workers per community	Rural communities: two trained workers per 5,000 population	Hard to reach areas: one trained worker per 1,000 population
Training	Rapid 3-day cascade training with limited attention to clinical practice	Incremental scale-up of 5-day training with attention to quality criteria	Incremental scale-up of 5-day training with attention to quality criteria
Medicines	Provision and supply against costs	Free	Free
Supervision	Irregular and mostly without clinical observation	Regular but infrequent clinical observation	Irregular and mostly without clinical observation
Demand creation	No specific approach	Part of routine activities of the female health army	Community leaders engaged in creation and management of village health clinics
Linkage with health facilities	Provider initiated	Weekly contacts	Monthly contacts
Monitoring	Tracking of program rollout	Tracking of program rollout	Tracking of program rollout
	Quality of care survey at end of project implementation	Tracking of indicators of implementation strength	Quality of care survey in year 2 of implementation
		Quality of care survey in year 2 of implementation	Introduction of implementation strength indicators in year 3
Review	Annual stakeholders meeting	Annual stakeholders meeting	Annual stakeholders meeting

CHWs = community health workers; iCCM = integrated community case management.
